# Variation Among Japanese Miso Breweries in Indoor Microbiomes is Mainly Ascribed to Variation in Type of Indoor Surface

**DOI:** 10.1007/s00284-023-03591-8

**Published:** 2024-01-18

**Authors:** Roger T. Koide, Makoto Kanauchi, Yasushi Hashimoto

**Affiliations:** 1 Department of Biology, Brigham Young University, Provo, UT USA; 2 Department of Food Management, Miyagi University, Sendai, Japan; 3 Section of Ecology and Environmental Science, Obihiro University of Agriculture and Veterinary Medicine, Obihiro, Japan

## Abstract

Miso is a microbially-fermented soybean food. The miso brewery indoor microbiome contributes to miso fermentation. Japanese breweries are not climate-controlled, so indoor spaces are strongly affected by the prevailing climate. Because climate influences microorganism distribution, our first hypothesis is that latitude, as a proxy for climate, is a major determinant of brewery indoor microbiome structure. Breweries vary in interior surface materials and in the way operations (steaming, processing, fermenting) are apportioned among rooms. Therefore, our second hypothesis is that more variability in indoor microbiomes exists among breweries than can be ascribed to a latitudinal gradient. Most miso produced today is inoculated with commercial microbial strains to standardize fermentation. If commercial strains outcompete indigenous microbes for membership in the indoor microbiome, this practice may homogenize indoor microbiomes among regions or breweries. Therefore, our third hypothesis is that inoculant fungal species dominate indoor fungal communities and make it impossible to distinguish communities among breweries or across their latitudinal gradient. We tested these hypotheses by sampling indoor surfaces in several breweries across a latitudinal gradient in Japan. We found that latitude had a significant but relatively small impact on indoor fungal and bacterial communities, that the effect of brewery was large relative to latitude, and that inoculant fungi made such small contributions to the indoor microbiome that distinctions among breweries and along the latitudinal gradient remained apparent. Recently, the Japanese Ministry of Agriculture, Forestry and Fisheries specified fungal inoculants to standardize miso production. However, this may not be possible so long as the indoor microbiome remains uncontrolled.

## Introduction

Miso is a fermented soybean product that has been an important food in Japan since the Yayoi period, 300 BC to 300 AD [1]. Miso production developed in Japan on a small scale, largely as a home-based industry [2] and, even now, much of the commercial miso production occurs in a multitude of small breweries [3]; in 2008 there were an estimated 1200 miso breweries throughout Japan [1]. Consequently, there are many varieties of miso that have resulted in local taste preferences [4].

Japanese miso is prepared by fermenting salted, steamed soybeans via the action of fungi and bacteria. Frequently, grains such as rice, wheat or barley are important additional components of miso [3, 4]. Traditionally, miso was not intentionally inoculated but was naturally colonized by indigenous fungi and bacteria circulating in the air and established on indoor surfaces [2]. Some refer to this traditional style of miso as “natto miso”, not to be confused with natto. In contrast, most modern miso is intentionally inoculated with an inoculum called “koji”, consisting of *Aspergillus oryzae* or *A. sojae* growing on steamed rice, barley or soybeans [2]. Specific strains of *A. oryzae* or *A. sojae* are chosen for their particular expression profiles of amylases, peptidases and proteases, and are usually purchased from a handful of Japanese suppliers [1]. Some brewers also intentionally inoculate their miso with the yeast, *Zygosaccharomyces rouxii* [5] and a lactic acid bacterium such as *Tetragenococcus halophilus* [3]. In 2021, the Japanese Ministry of Agriculture, Forestry and Fisheries proposed rules to further standardize miso quality, specifying *Aspergillus oryzae* as the only fermenting microorganism and listing the permissible major ingredients for four categories of miso [6].

Despite the intentional inoculation of miso with only one or two fungal strains and possibly a single bacterium, the microbial community of finished, commercially-produced miso is surprisingly complex, frequently comprising dozens of fungal and bacterial species [2, 4, 7–10]. The reason for this microbial diversity stems from the unintentional inoculation of miso by airborne propagules of microbes comprising the indoor microbiome, those that are indigenous to the area and that have established themselves on interior brewery surfaces [2]. Many miso brewers consider this unintentional inoculation of miso by the indoor microbiome to make an essential contribution to the quality of the finished product. As with wine production [11, 12], the species composition of the fungi and bacteria present in miso significantly influences its chemical composition and, therefore, its flavor, aroma and visual characteristics [5, 8, 13].

Grape skins are not sterilized prior to wine fermentation. Because the grape skin microbiome contributes significantly to wine fermentation [14], the indoor microbiome becomes less consequential to wine fermentation. In contrast, miso ingredients are steamed prior to fermentation and are, therefore, sterile or nearly sterile. Because there is essentially no microbiome associated with steamed soybeans, the indoor microbiome is functionally far more consequential to miso fermentation. Therefore, some brewers will go to great lengths to maintain consistency in the brewery indoor microbiome. For example, the wooden beams and wall panels from an old miso brewery, as well as the staves of disused miso fermentation barrels, all of which are naturally colonized by communities of fungi and bacteria, are frequently salvaged and installed in a new brewery to establish the mature, indoor microbiome of the old brewery.

The geographical structure of microbial communities, or microbial biogeography [11], may be at least partly responsible for the terroir of wine [11, 12] and, similarly, for regional differences in the qualities of miso [15]. This is possible because most miso breweries in Japan are neither air-conditioned in summer nor heated in winter, so they experience conditions that reflect the prevailing climate. Microbial biogeography exists because the distribution of microorganisms is, in part, determined by climatic factors such as temperature and moisture [16–19]. It is not surprising, therefore, that climate is known to influence the composition of microbial communities involved in the production of fermented foods [20–22]. Therefore, our first hypothesis is that latitude, as proxy for climate [11], is a major determinant of the structure of the brewery indoor microbiome.

Miso breweries throughout Japan differ from each other in a number of ways. The operations necessary for miso production (steaming, processing [mixing ingredients and packing into bins or barrels], fermenting) are apportioned among interior spaces differently in different breweries. For example, in small breweries all operations may occur in a single room while in others, each operation may occur in a separate room (Table 1). Furthermore, indoor brewery surfaces (walls, ceilings, fermentation vats, etc.) may be made from different materials, including wood, concrete, plastic and metal. Because operations and construction materials undoubtedly influence the indoor microbiome [23], individual breweries may differ in their indoor microbiomes irrespective of climate and latitude. Our second hypothesis, therefore, is that there is more variability among brewery indoor microbiomes than exists along their latitudinal gradient.

**Table 1:** Miso breweries, locations, surfaces sampled, rooms sampled, and daily average maximum and minimum air temperatures from 14 June–14 July 2022

Date of sampling	Miso Brewery	City, Prefecture	Surface/Room^a^	Decimal latitude, longitude	Daily max T (°C)	Daily min T (°C)
12 July 2022	Koizumi Miso	Ōsaki, Miyagi	Mixed/Processing	38.64924913025727, 140.86544384572846	27.4	19.9
11 July 2022	Oota Yohachiro Miso	Shiogama, Miyagi	Wood/Proc.-Ferm	38.31787841482078, 141.01957212289085	27.3	19.7
4 July 2022	Horaiya Honten Co., Ltd	Koriyama, Fukushima	Smooth/Processing	37.37359067546357, 140.39624445362946	24.2	16.5
20 July 2022	Nihon Miso	Yokohama, Kanagawa	Concrete/Fermenting	35.49114903226308, 139.59959458794293	30.1	23.9
6 July 2022	Isoda Daitokuji Natto Miso	Kyoto, Kyoto	Smooth/Outdoors	35.04488939109675, 135.74681049748625	28.9	21.6
6 July 2022	Kyoto Kokonoe Miso	Otsu, Shiga	Wood/Proc.-Ferm	35.00956035641118, 135.86358418300605	28.9	21.6
15 July 2022	Noda Miso	Toyota, Aichi	Wood/Fermenting	34.9986855949468, 137.13816575469667	31.4	23.1
14 July 2022	Ichibiki Co., Ltd. (1)	Toyokawa, Aichi	Wood/Fermenting	34.84487431226333, 137.31606864665406	28.5	23.2
14 July 2022	Ichibiki Co., Ltd. (2)	Toyokawa, Aichi	Smooth/Steaming	34.84487431226333, 137.31606864665406	28.5	23.2
14 July 2022	Kunimatsu Honten	Toyohashi, Aichi	Mixed/Proc.-Ferm	34.77408714442065, 137.3821571195508	28.5	23.2
8 July 2022	Tashimaya Miso (1)	Fukuoka, Fukuoka	Smooth/Processing	33.66869051178875, 130.44198969406995	29.7	23.5
8 July 2022	Tashimaya Miso (2)	Fukuoka, Fukuoka	Smooth/Fermenting^b^	33.66869051178875, 130.44198969406995	30.0	30.0

^a^Mixed Surface = Wood & Smooth, Smooth = plastic or painted metal, Processing = the room in which steamed soybeans, salt and koji are mixed and packed into bins or barrels for subsequent fermentation. Proc.-Ferm. = the room in which both processing & fermenting occur, Steaming = room in which soybean steaming occurs; ^b^This room was controlled at 30 °C. All other sampled rooms were not temperature-controlled

The common use of standard, commercially-available microbial strains for miso fermentation has undoubtedly promoted consistency and reliability in the fermentation process just as it has for wine fermentation [23], but it may also homogenize region-specific or brewery-specific indoor microbiomes and may thus attenuate local distinctions in miso quality as it has for wine terroir [11, 23]. Our third hypothesis, therefore, is that inoculant fungal species dominate the indoor fungal communities and thus make it impossible to distinguish fungal communities among breweries or across their latitudinal gradient.

We tested the three hypotheses by sampling indoor surfaces in miso breweries from 38.65 to 33.67 degrees N latitude (Fukushima to Fukuoka) along the Japanese archipelago (Table 1) and analyzing the fungal and bacterial communities based on high-throughput DNA sequencing.

## Materials and Methods

### Brewery sampling

We sampled miso breweries between 4 and 20 July 2022 (Table 1). Where permitted (at most miso breweries), we took 18 samples from the dominant interior surface. There were two exceptions: Kunimatsu Honten, where only 5 interior surface samples were taken, and Isoda Daitokuji Natto Miso, where we were not allowed to sample the interior of the brewery and where, for comparative purposes, we sampled from an outdoor fence immediately adjacent to the brewery. We sampled all environmental surfaces using sterile collection swabs (#C1100-20, Zymo Research, Irvine, CA, USA) by rotating the swab tip along an approximately 10 cm length of the surface. After sampling, the swab tips were broken off into sterile, 2 ml microcentrifuge tubes to which we added 1 ml of DNA/RNA Shield™ (Zymo Research, Irvine, CA, USA) for storage at room temperature until taken to Brigham Young University for processing.

Depending on the brewery, we sampled from the dominant interior surface type, which was either unpainted, coarse-textured wood (Wood) or unpainted, coarse-textured concrete (Concrete), or a plastic or painted metal surface (Smooth). In the Koizumi brewery, half of the samples were taken from wooden surfaces and half from smooth surfaces because there was no single dominant surface type in the room. In that case, we characterized the indoor surface at that brewery as “Mixed”. Depending on the brewery, various miso production steps (soybean steaming, miso processing [mixing of ingredients and packing into bins or barrels for subsequent fermentation], and miso fermentation), were carried out in one, two or three separate rooms. When permitted, we sampled in the room used for miso processing because this was the room in which unintended inoculation of miso from airborne microbial propagules was most likely. In some cases, we were permitted to sample only in the room used for miso fermentation (Table 1). In some breweries, both processing and fermentation occurred in the same room. At each of two breweries (Ichibiki, Tashimaya), we sampled two rooms. As indicated above, at Isoda Daitokuji Natto Miso, we sampled outdoors. None of the rooms were temperature-controlled except for the fermentation room at Tashimaya brewery (“Tashimaya Miso 2,” Table 1).

Upon return to Brigham Young University on 22 July 2022, samples were refrigerated at 5 °C for no longer than 2 weeks prior to DNA extraction.

### DNA Extraction

We used a modification of the Quick-DNA Fungal/Bacterial Miniprep kit method for DNA extraction (Zymo Research). The BashingBeads from a ZR BashingBead Lysis Tube were emptied into the 2 ml microcentrifuge tube from Japan containing the sampling swab tip and 1.0 ml DNA/RNA Shield. This was shaken for 6 min. at 1200 RPM on a 2010 Geno/Grinder (SPEX SamplePrep, Metuchen, NJ, USA), followed by the standard DNA cleanup provided by the Quick-DNA Fungal/Bacterial Miniprep kit.

### MiSeq Library Preparation

#### Fungal Thermal Cycling

All extracted DNA samples were subjected to a two-step PCR amplification with PCRBIO Hot Start VeriFi master mix (Genesee Scientific, El Cajon, CA, USA). In the first step, the ITS1 region was amplified using the primers ITS1f [24] and ITS2 [25] appended to sequences CS1 (*5′-*ACACTGACGACATGGTTCTACA*-*3′) and CS2 (5′-TACGGTAGCAGAGACTTGGTCT-3′), respectively. The first thermal cycling program was: hot-start activation at 95 °C for 1 min, 27 cycles of 95 °C for 15 s, 60 °C for 15 s, and 72 °C for 18 s, with final elongation at 72 °C for 10 min. In the second step, barcodes and Illumina flowcell adapters were appended to the PCR1 amplicons. The second thermal cycling program was: hot-start activation at 95 °C for 1 min, 12 cycles of 95 °C for 15 s, 55 °C for 30 s, and 68 °C for 40 s with final elongation at 68 °C for 10 min.

#### Bacterial Thermal Cycling

All extracted DNA samples were subjected to a two-step PCR amplification with PCRBIO Hot Start VeriFi master mix. In the first thermal cycling program, the V4 region was amplified using 515f and 806r primers [26] appended to sequences CS1 and CS2, respectively using the same program as for the fungi. In the second program, barcodes and Illumina flowcell adapters were appended to the PCR1 amplicons with the same program used for the fungal libraries.

#### Library Preparation and Sequencing

Identical volumes of the PCR2 products from each of the samples were pooled to create separate fungal and bacterial sequencing libraries. Sequencing was done at the Institute for Bioinformatics and Evolutionary Studies (iBEST) genomics resources core at the University of Idaho, Idaho Genomics Resources Core (http://www.ibest.uidaho.edu/, Moscow, ID). At iBest, libraries were quantified with Qubit and subjected to fragment analysis for quality control. The libraries were then bead-cleaned and rechecked on the fragment analyzer for quality. Amplicon libraries were sequenced using 2 × 300 paired-end reads on an Illumina MiSeq v3 (600 cycles) sequencing platform (Illumina Inc., San Diego, CA, USA).

#### Bioinformatics

Reads were filtered using the DADA2 pipeline [27] version 1.16. We trimmed reads with quality scores less than 10 (truncQ = 10) and filtered out reads less than 150 bp in length. Chimeras were removed by comparison against the Gold database. Sequences were clustered at 97% similarity. Taxonomy was assigned using the UNITE database for fungi and the Silva database for bacteria and the Ribosomal Database Project Naïve Bayesian Classifier algorithm [28] with kmer size of 8, and 50% bootstrap threshold required to assign taxonomy. To compare samples at equivalent sequencing depth, all fungal samples were rarefied to 94 reads per sample, which eliminated 3 samples from the fungal library, and all bacterial samples were rarefied to 229 reads per sample, which eliminated 2 samples from the bacterial library.

#### Statistical Analyses

﻿To compare fungal or bacterial communities among breweries, surfaces, and rooms (see Table 1), we performed permutational multivariate analyses of variance (PERMANOVA) in the R statistical environment [29] with the vegan package [30] using Bray–Curtis dissimilarities [31]. For the period of 14 June to 14 July 2022, the averages of the daily minimum and maximum air temperatures were calculated using data from weather.com for the cities in which the breweries were located. Variation in community structure was visualized as a two-dimensional ordination (non-metric multidimensional scaling, NMDS) and Bray–Curtis dissimilarities using the vegan package. Environmental variables (minimum and maximum air temperatures, latitude) were fitted onto the NMDS plots using the envfit function in vegan.

We also characterized differences among rooms, surface types and breweries with respect to the rarefied frequencies of the most frequently occuring fungal and bacterial taxa. ﻿Because both bacterial and fungal communities were composed of hundreds of individual taxa, we limited these frequency analyses to the taxa contributing to at least 2% of all fungal reads or at least 1% of all bacterial reads. Thus, out of a total of 259 total bacterial taxa, the 18 most common bacterial taxa were subject to this analysis, and, out of a total of 205 total fungal taxa, the 11 most common fungal taxa were analyzed. We analyzed the rarefied frequency of occurrence of the common fungal and bacterial taxa according to room, surface type and brewery by building generalized linear models with negative binomial distributions for each of the taxa. Models were built using the DESeq2 package [32] in the R environment. A negative binomial distribution was chosen as it has been used frequently to accurately describe microbial abundances [32, 33], which are often non-normally distributed and difficult to model using common statistical models such as ANOVA. To protect against false positives, all P values from these models were adjusted according to Benjamini and Hochberg [34].

## Results

### Fungal communities

**Fig. 1:**
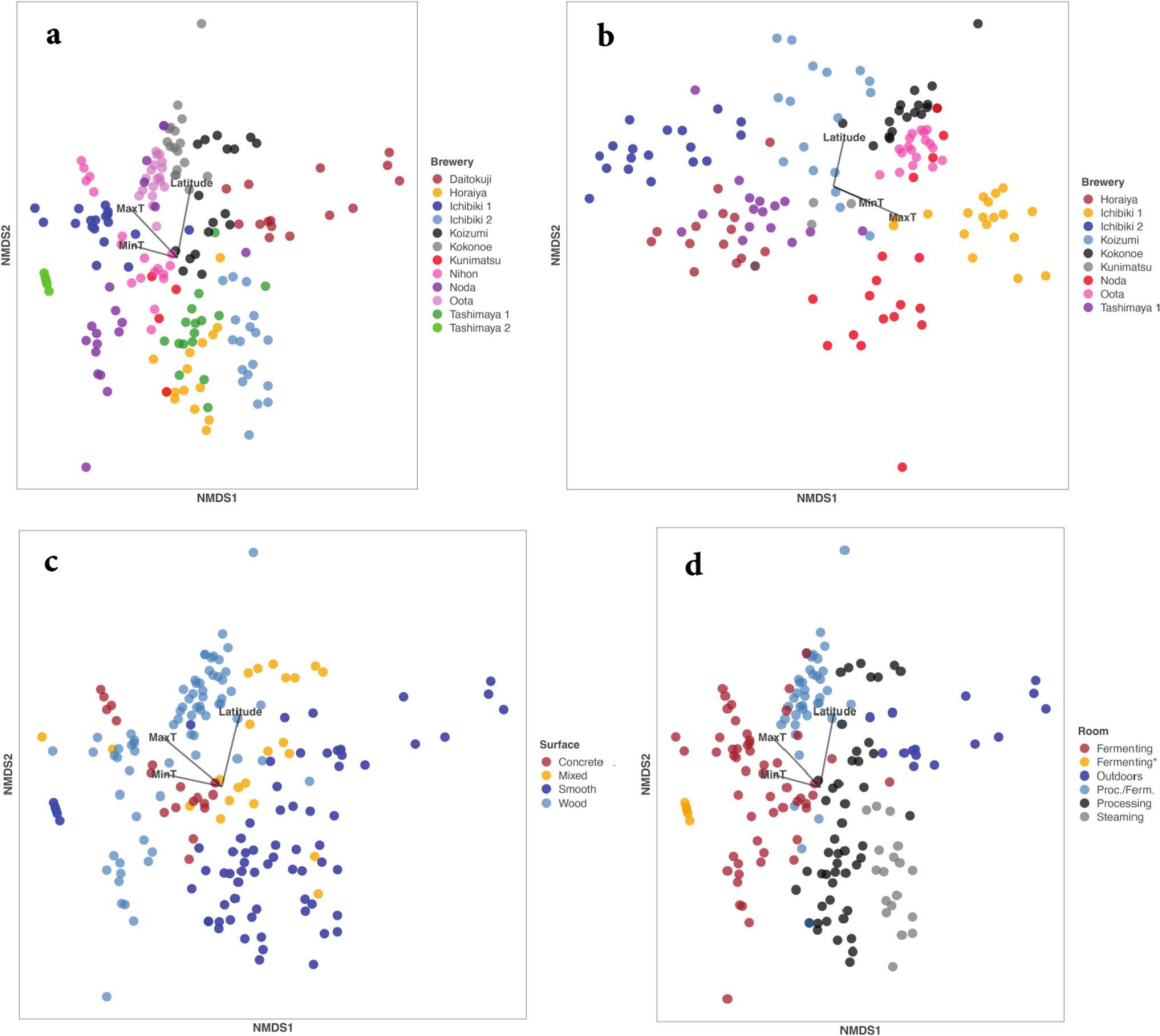
**a** NMDS plot of fungal communities by brewery. Vectors for environmental variables (Minimum T, Maximum T and Latitude, see materials and methods) are also plotted. For details of the breweries, see Table 1. **b** NMDS plot of fungal communities by brewery. The Daitokuji, Tashimaya2 and Nihon breweries were eliminated from this analysis because they were sampled outdoors, in a controlled-temperature room, or in an underground room, respectively. All remaining breweries included in this analysis were sampled from rooms that were not temperature-controlled. Vectors for environmental variables (Minimum T, Maximum T and Latitude, see materials and methods) are also plotted. **c** NMDS plot of fungal communities by surface. **d** NMDS plot of fungal communities by room. The asterisk indicates samples from Tashimaya Miso (2) fermentation room, which is the only controlled-temperature room (30 °C) in the study

Considering all breweries, latitude was a statistically significant factor, but it accounted for only 5% of the total variation in Bray–Curtis distance, the measure of the difference in fungal community structure. For simplicity, we hereafter refer to the variation in Bray–Curtis distance as the variation in community structure. MaxT was also a significant factor, accounting for 7% of variation, as was MinT, accounting for 4% (Table 2, Fig. 1a). Daitokuji Natto Miso brewery was sampled outdoors so those samples do not necessarily reflect the indoor microbiome at that brewery. Tashimaya2 samples came from a controlled-temperature room, and Nihon brewery was sampled in an underground room, so these two were assumed to be influenced less by the prevailing climate than the other breweries, which were sampled in rooms that were not temperature-controlled. Therefore, Daitokuji, Tashimaya2 and Nihon breweries were eliminated in a second analysis. However, this resulted in only a slight change in the result; latitude was, again, significant, accounting for 7% of the variation in fungal community structure, while MaxT was significant, accounting for 10%, and MinT was no longer significant (Table 3, Fig. 1b).

**Table 2:** The contribution of the environmental variables (latitude, maximum and minimum air temperatures) to the structure of fungal communities. See Figs. 1a-c. r^2^ is the variation explained by the various environmental factors in the multiple regression model

	r^2^	P
Latitude	0.0512	0.007
MaxT	0.0659	0.001
MinT	0.0447	0.016

**Table 3:** The contribution of the environmental variables (latitude, maximum and minimum air temperatures) to the structure of fungal communities. The Daitokuji, Tashimaya2 and Nihon breweries were eliminated from this analysis because they were sampled outdoors, in a controlled-temperature room, or in an underground room, respectively. All remaining breweries included in this analysis were sampled from rooms that were not temperature-controlled. See Fig. 1d. r^2^ is the variation explained by the multiple regression model

	r^2^	P
Latitude	0.0687	0.010
MaxT	0.1032	0.001
MinT	0.0304	0.128

Compared to latitude, brewery accounted for a much greater percentage of the variation (47% of total) in fungal community structure (Table 4, Fig. 1a), while Surface (17%, Table 5, Fig. 1c) and room (31%, Table 6, Fig. 1d) also accounted for significant but lower percentages of variation.

**Table 4:** Permanova results for fungal communities by Brewery

	df	SS	MS	F	r^2^	P
Brewery	11	38.035	3.4577	13.957	0.47308	1.00E-04
Residuals	171	42.363	0.2477		0.52692	
Total	182	80.398			1.00000

**Table 5:** Permanova results for fungal communities by Surface

	df	SS	MS	F	r^2^	P
Surface	3	13.681	4.5604	12.261	0.17047	1.00E-04
Residuals	179	66.575	0.3719		0.82953	
Total	182	80.256			1.00000

**Table 6:** Permanova results for fungal communities by Room

	df	SS	MS	F	r^2^	P
Room	5	24.527	4.9055	15.58	0.30562	1.00E-04
Residuals	177	55.728	0.3148		0.69438	
Total	182	80.256			1.00000	

**Fig. 2:**
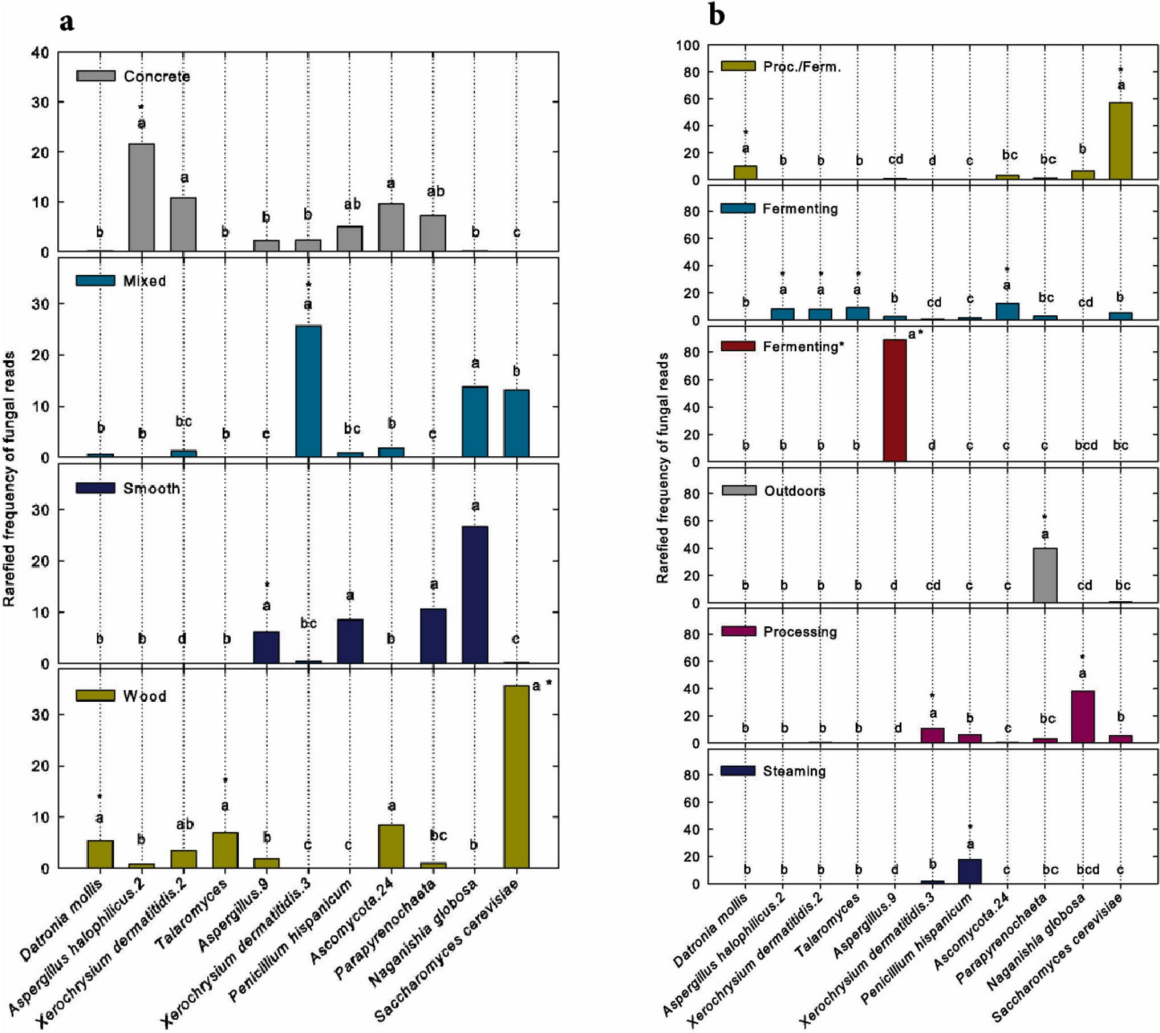
**a** Rarefied frequencies of sequence reads of the 11 most frequently occurring fungal taxa on each of the surfaces. The frequencies were analyzed by building generalized linear models with negative binomial distributions for each of the taxa. Different letters indicate significant differences among surfaces in the frequencies of a given taxon. To protect against false positives, all P values were Benjamini–Hochberg adjusted. Asterisks indicate taxa that occurred most frequently on a particular surface. **b** Rarefied frequency of reads of the 11 most frequently occurring fungal taxa within each of the rooms. The frequencies were analyzed as in **a**. Different letters and asterisks indicate the same as in **a**. None of the rooms were temperature-controlled except for the fermenting room at Tashimaya brewery, marked with an asterisk, which was controlled at 30 °C

Variation in fungal community structure among surfaces was reflected in variation among surfaces in the rarefied read numbers of particular fungal taxa, which are proportional to relative taxon frequencies (Fig. 2a). For example, *Aspergillus halophillicus.*2 occurred most frequently on concrete, *Xerochrysium dermatitidis* occurred most frequently on mixed surfaces, *Aspergillus.*9 occurred most frequently on smooth surfaces, and *Datronia mollis, Talaromyces* and *Saccharomyces cerevisiae* occurred most frequently on wood. The taxon identifications, including the numerals, are those given in the UNITE database.

Variation in fungal community structure among rooms was also reflected in variation among rooms in the rarefied read numbers of particular fungal taxa, which are proportional to relative taxon frequencies (Fig. 2b). For example, *Datronia mollis* and *Saccharomyces cerevisiae* occurred most frequently in combination processing/fermentation rooms, *Aspergillus halophillicus*.2, *Xerochrysium dermatitidis*.2, *Talaromyces* and Ascomycota.24 occurred most frequently in fermentation rooms (not temperature controlled), *Aspergillus*.9 occurred most frequently in the temperature-controlled fermentation room, *Parapyrenochaeta* occurred most frequently outdoors (labeled “neither”), *Xerochrysium dermatitidis*.3 and *Naganishia globosa* occurred most frequently in processing rooms, and *Penicillium hispanicum* occurred most frequently in the soybean steaming room.

While the inoculant fungus (genus *Aspergillus*) occurred in every brewery, it was not a major component of fungal communities on any particular surface (Fig. 2a) or in any particular room, except in the temperature-controlled fermenting room of the Tashimaya brewery (Fig. 2b).

Bacterial communities.

**Fig. 3:**
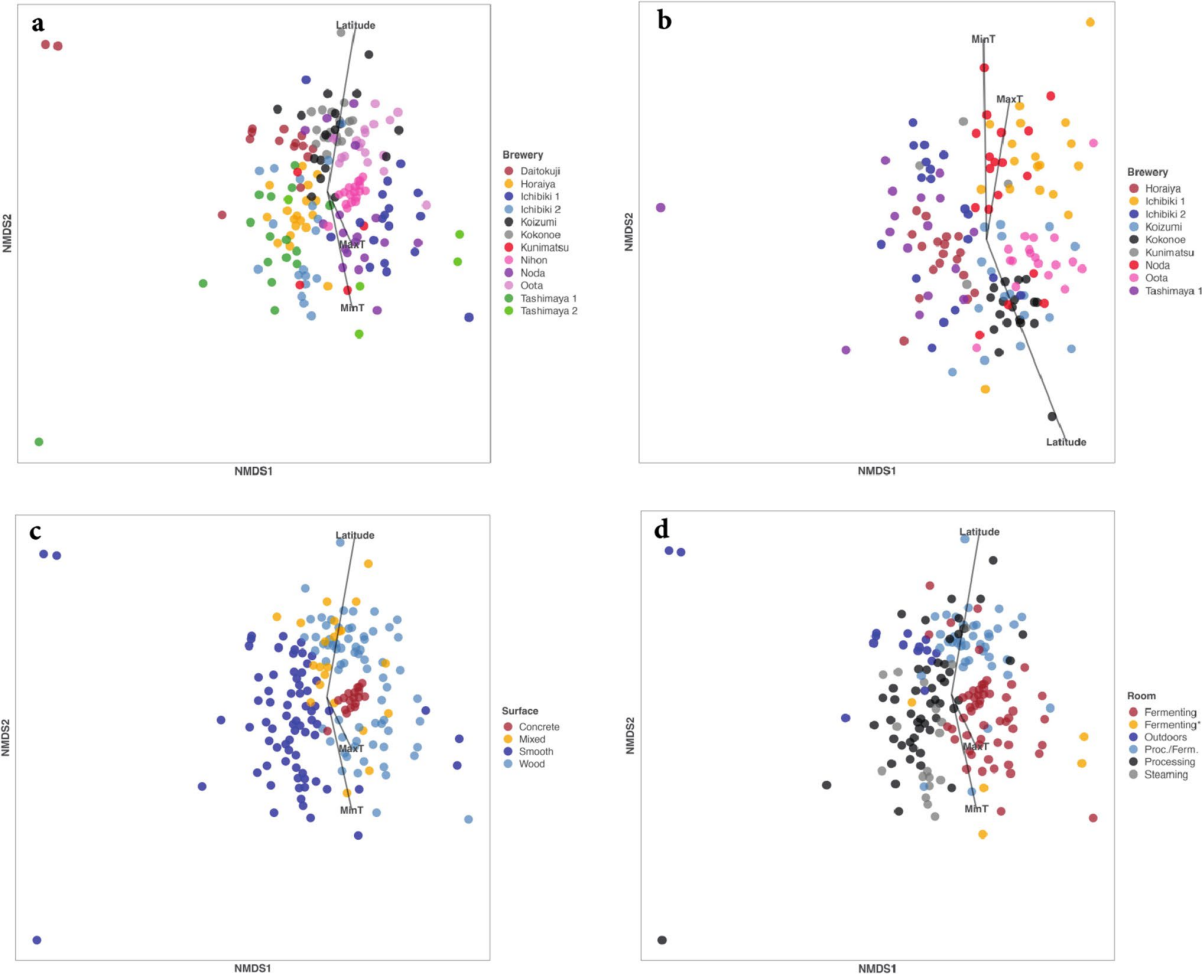
**a** NMDS plot of bacterial communities by brewery. Vectors for environmental variables (Minimum T, Maximum T and Latitude, see materials and methods) are also plotted. For details of the breweries, see Table 1. **b** NMDS plot of bacterial communities by brewery. The Daitokuji, Tashimaya2 and Nihon breweries were eliminated from this NMDS because they were sampled outdoors, in a controlled-temperature room, or in an underground room, respectively. All remaining breweries included in this analysis were sampled from rooms that were not temperature-controlled. Vectors for environmental variables (Minimum T, Maximum T and Latitude, see materials and methods) are also plotted. For details of the breweries, see Table 1. **c** NMDS plot of bacterial communities by surface. **d** NMDS plot of bacterial communities by room

Latitude was a significant factor and accounted for 19% of variation in bacterial community structure. MaxT was not a significant factor, but MinT was significant and accounted for 10% of variability in bacterial community structure (Table 7, Fig. 3a). Daitokuji Natto Miso brewery was sampled outdoors so those samples do not reflect an indoor microbiome. Tashimaya2 samples came from a controlled-temperature room, and Nihon brewery was sampled in an underground room, so these two were assumed to be controlled less by the prevailing climate than the other breweries, which were sampled in rooms that were not temperature-controlled. Therefore, Daitokuji, Tashimaya2 and Nihon breweries were eliminated in a second analysis. However, this resulted in only a slight change in the result; latitude was significant and accounted for 20% of the variation in bacterial community structure, MaxT was significant and accounted for 8% and MinT was significant and accounted for 14% (Table 8, Fig. 3b).

**Table 7:** The contribution of the environmental variables (latitude, maximum and minimum air temperatures) to the structure of bacterial communities. See Fig. 3a. r^2^ is the variation explained by the multiple regression model

	r^2^	P
Latitude	0.1887	0.001
MaxT	0.0262	0.088
MinT	0.0954	0.001

**Table 8:** The contribution of the environmental variables (latitude, maximum and minimum air temperatures) to the structure of bacterial communities. The Daitokuji, Tashimaya2 and Nihon breweries were eliminated from this analysis because they were sampled, respectively, outdoors, in a controlled-temperature room, or in an underground room. All remaining breweries included in this analysis were sampled from rooms that were not temperature-controlled. See Fig. 3b. r^2^ is the variation explained by the multiple regression model

	r^2^	P
Latitude	0.2033	0.001
MaxT	0.0756	0.005
MinT	0.143	0.001

Compared to latitude, brewery accounted for a much greater percentage of the variation (45% of total) in bacterial community structure (Table 9, Fig. 3a). Surface (16%, Table 10, Fig. 3c) and room (20%, Table 11, Fig. 3d) also accounted for significant but lower percentages of the variation.

**Table 9:** Permanova results for bacterial communities by Brewery

	df	SS	MS	F	r^2^	P
Brewery	11	35.626	3.2387	12.497	0.44564	1.00E-04
Residuals	171	44.317	0.2592		0.55436	
Total	182	79.942			1.00000

**Table 10:** Permanova results for bacterial communities by Surface

	df	SS	MS	F	r^2^	P
Surface	3	13.114	4.3714	11.709	0.16405	1.00E-04
Residuals	179	66.828	0.3733		0.83595	
Total	182	79.942			1.00000

**Table 11:** Permanova results for bacterial communities by Room

	df	SS	MS	F	r^2^	P
Room	5	15.883	3.1767	8.7775	0.19869	1.00E-04
Residuals	177	64.059	0.3619		0.80131	
Total	182	79.942			1.00000	

**Fig. 4:**
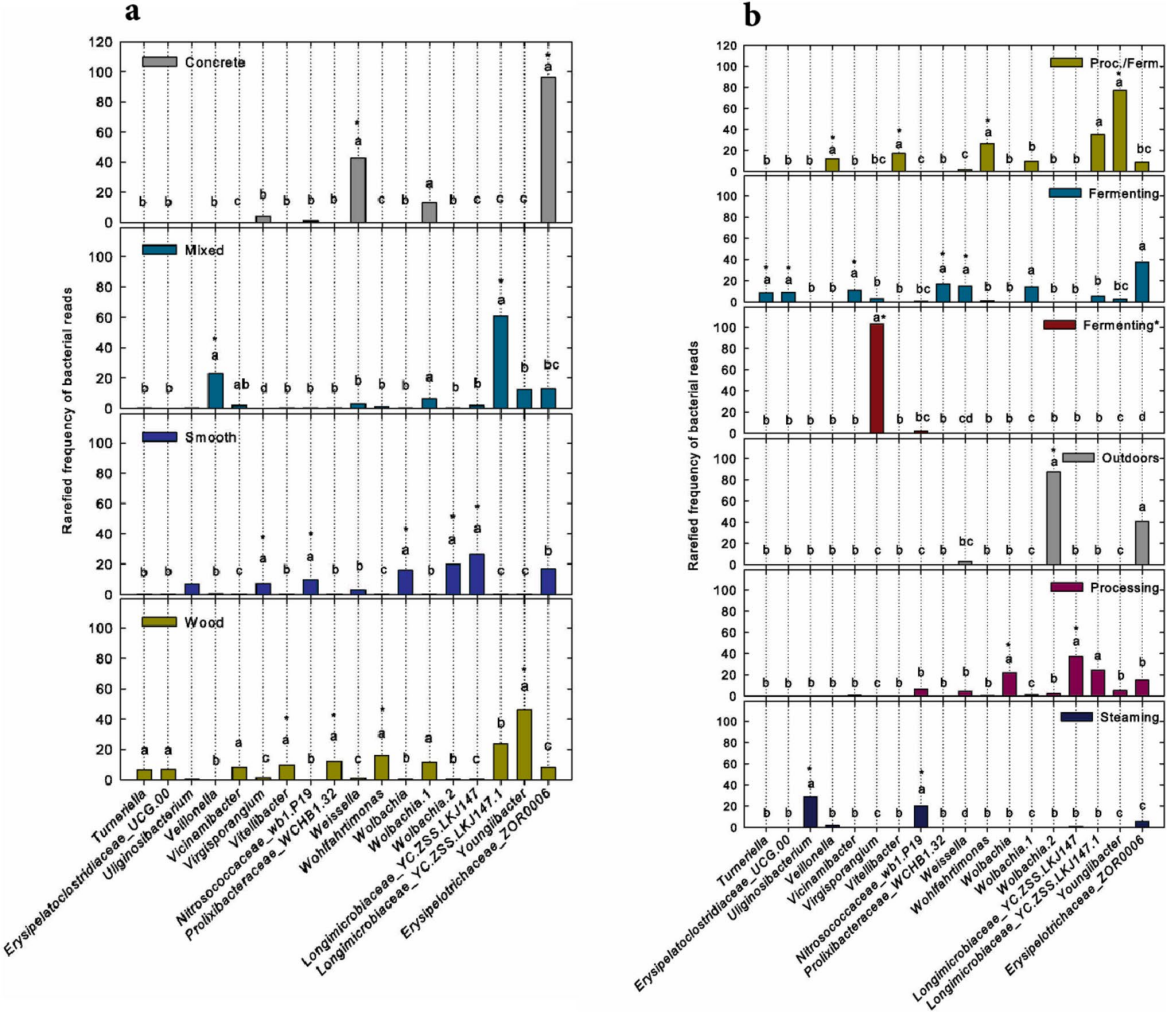
**a** Rarefied frequencies of reads of the 18 most frequently occurring bacterial taxa on each of the surfaces. The frequencies were analyzed by building generalized linear models with negative binomial distributions for each of the taxa. Different letters indicate significant differences among surfaces in the frequencies of a given taxon. To protect against false positives, all P values were Benjamini–Hochberg adjusted. Asterisks indicate taxa that occurred most frequently on a particular surface. The frequency of *Uliginosibacterium* did not vary significantly among surfaces. **b** Rarefied frequencies of reads of the 18 most frequently occurring bacterial taxa within each of the rooms as in **a**

Variation in bacterial community structure among surfaces was reflected in variation among surfaces in the rarefied read numbers of particular bacterial taxa, which are proportional to relative taxon frequencies (Fig. 4a). For example, *Weissella sp.* and *Erysipelotrichaceae_*ZOR0006 occurred most frequently on concrete, *Veillonella sp.* and *Longimicobiaceae_*YC.ZSS.LKJ147.1 occurred most frequently on mixed surfaces, *Virgisporangium, Nitrosococcaceae_*wb1.P19*, Wolbachia, Wolbachia.*2 and Longimicrobiaeae*_*YC.ZSS.LKJ147 occurred most frequently on smooth surfaces, and *Vitellibacter, Prolixibacteraceae_*WCHB1.32*, Wohlfahrtimonas* and *Youngibacter* occurred most frequently on wood (Fig. 4a). The taxon identifications, including the numerals, are those given in the Silva database.

Variation in bacterial community structure among rooms was also reflected in variation among rooms in the rarefied read numbers of particular bacterial taxa, which are proportional to relative taxon frequencies (Fig. 4b). For example, *Veillonella, Vitellibacter, Wohlfahrtimonas and Youngiibacter* occurred most frequently in combination rooms, *Turneriella*, Erysipeiatoclostridiaceae_UCG.00, *Vicinamibacter*, Prolixibacteraceae_WCHB1.32 and *Weissella* occurred most frequently in fermentation rooms (not temperature controlled), *Virgisporangium* occurred most frequently in the temperature-controlled fermentation room, *Wolbachia*.2 occurred most frequently outdoors (labeled “neither”), *Wolbachia* and Longimicrobiaceae_YC.ZSS.LKJ147 occurred most frequently in processing rooms, and *Uliginosibacterium* and Nitrosococcaceae_wb1.P19 occurred most frequently in the soybean steaming room.

## Discussion

Latitude is highly correlated with climate [11] and controls the distribution of various organisms along the length of the Japanese archipelago, including warm-adapted vs. cool-adapted plant species [35]. Microorganisms also exhibit a biogeography that is influenced by climatic factors such as temperature [16–19]. Because Japanese miso breweries are generally neither air conditioned in summer nor heated in winter, our first hypothesis was that latitude would be largely responsible for variation in their indoor microbiomes. Latitude was a significant factor for both indoor brewery fungal and bacterial communities, but it explained only 7% of the variation in fungal community structure and 20% of the variation in bacterial community structure. Mean daily minimum and maximum air temperatures during the month prior to sampling were also significant with respect to microbial community structure. For bacterial communities, the temperature vectors were generally parallel but directionally opposite to the latitude vector, as expected; increasing temperatures were generally associated with decreasing latitude. For the fungal communities, however, the temperature vectors were largely orthogonal to the latitude vector, which was unexpected. This may have occurred because, in the structuring of fungal communities, factors other than temperature contributed to the latitude effect. Thus, while a case could be made for some degree of regional variation in miso quality [15] as a consequence of the biogeography of the indoor brewery microbiome, just as it has been made for wine terroir [11, 12], such an effect due to latitude did not appear to be large. Nevertheless, the small but significant effect of latitude and temperature on the indoor brewery microbiome suggests that climate change may have an effect on the indoor microbiome and, therefore, on miso fermentation.

Our second hypothesis was that major differences occur among individual breweries in their indoor microbiomes, irrespective of latitude. Indeed, variation among breweries was large for both fungal and bacterial communities, respectively contributing 47% and 45% of the total variation in community structure, irrespective of latitude. Thus, intrinsic differences among breweries appear to be far more important than latitude.

Breweries differed from each other in both the operations that occurred within the rooms we were allowed to sample as well as in the nature of the surfaces within those rooms. Surface had a significant impact, accounting for 17% of the variation in fungal community structure and 16% of the variation in bacterial community structure. For both fungi and bacteria, smooth (plastic or painted metal) surfaces and coarse-textured wood surfaces harbored very distinct communities, while mixed (smooth and wood) surfaces harbored communities of intermediate composition. The fungal and bacterial communities on the coarse-textured concrete were more similar to those on wood than they were to those on smooth surfaces. These results suggest that texture (smooth vs. porous) is an important variable with respect to microbial community structure.

For both fungi and bacteria, taxon frequencies differed significantly among surfaces such that different surfaces could be characterized by high frequencies of distinguishing taxa. For example, we found *Datronia mollis,* a wood-rotting basidiomycete [36], most frequently on wooden surfaces. *Saccharomyces cerevisiae,* brewer’s or baker’s yeast, which is not a wood-rotting fungus, was often but inexplicably also dominant on wooden surfaces. *Talaromyces* are common indoor fungi [37], and we found them most frequently also on wooden surfaces (in fermentation rooms). Members of the Restricti group of species within *Aspergillus*, such as *A. halophilicus*, are xerophilic and commonly found on dry building materials [38]. In our study *A. halophilicus* was most frequently found on concrete. *Xerochrysium dermatitidis* is xerophylic and associated with soy products [39]. We found it growing most frequently in breweries with a combination of smooth and wooden surfaces. *Vitellibacter* is a genus of salt-tolerant bacteria [40] that was preferentially associated with wooden surfaces in our study. The family Prolixibacteraceae is commonly found in saline coastal sediment [41]. In our study it was found mainly on wooden surfaces. *Weisella* has been found in fermenting moromi, immature soy sauce [42], which is similar to fermenting miso. We found *Weisella* primarily on concrete surfaces, but we have no explanation for this preference. Erysipelotrichaceae are found in the human intestine [43]. Their presence in miso breweries, especially on concrete, is currently inexplicable.

The particular room (differing in operation) in which we sampled accounted for 31% of the variation in fungal community structure and 20% of the variation in bacterial community structure. Even within a single brewery, different rooms had very distinct fungal and bacterial communities; such was the case within both the Ichibiki and Tashimaya breweries. For both fungi and bacteria, processing rooms and fermenting rooms harbored distinct communities, while combination (processing & fermenting) rooms harbored communities of somewhat intermediate composition, overlapping communities in both processing and fermenting rooms.

For both fungal and bacterial communities, taxon frequencies differed significantly among rooms such that different rooms could be characterized by high frequencies of distinguishing taxa. *Veillonella* is a genus of obligate anaerobic bacteria capable of utilizing carbohydrates [44]. Miso fermentation can provide an anaerobic or nearly anaerobic environment [45], so it is not too surprising to find this genus in miso breweries. In our study it was found almost exclusively in the soybean steaming room. *Virgisporangium* appears to be primarily a genus of soil actinomycetes. At least some strains are capable of growth at 40 °C [46]. It was found almost exclusively in the controlled-temperature (30 °C) fermentation room of the Tashimaya brewery. Nitrosococcaceae is a family of nitrifying bacteria [47] and, thus, expected in settings where high proteins materials, such as soybean, are fermented. Nitrosococcaceae were most frequently found in the soybean steaming room of the Ichibiki brewery. *Wolbachia* have reduced genomes and are fascinating endosymbionts of various invertebrates [48]. They were present almost entirely outdoors in our study.

Because the variation attributed to surface and room, although large, was still less than the variation attributed to brewery, it would appear that, in addition to room and surface, other environmental factors contribute strongly to the effect of brewery on indoor microbiome structure. We can only speculate as to what these factors might be, but suggestions include, for example, age of structure and proximity to vegetation [49].

The common use of standard, commercially available microbial strains in wine fermentation has promoted consistency and reliability in the fermentation process [23], but it has also reduced the potential for the development of regionally distinct terroir [11, 12]. Therefore, our third hypothesis was that in miso fermentation, the standard inoculant fungi (genus *Aspergillus*) dominate the indoor fungal communities to the extent that it makes it impossible to distinguish fungal communities among breweries or across a latitudinal gradient. While *Aspergillus* was a component of fungal communities in various rooms and on various surfaces, it occurred frequently in only one instance, the controlled-temperature fermenting room of the Tashimaya brewery. Thus, it does not appear that standard inoculant fungi have come to dominate the indoor fungal community despite their frequent use. The consequence of this is that it may still be possible to establish regionally-specific indoor fungal communities, which could contribute to regional differences in miso quality as suggested by Sun et al. [15]. However, if terroir does exist for miso, it does not appear to be strongly controlled by latitude.

Our study did not include characterization of the microbiome of finished miso for two major reasons. First, the miso microbiome undergoes a rapid succession during the fermentation process [10]. Therefore, while the indoor microbiome may have lasting impacts on miso quality, it may be distinguishable only transiently in the miso microbiome as has been shown for vineyard-specific grape microbiomes during wine fermentation [50]. Moreover, the miso microbiome is at least a partial function of the indoor microbiome as it interacts with miso composition, which varies widely from brewery to brewery [3]. Nevertheless, it is probable that indoor microbiomes do contribute to miso fermentation. The fungi commonly found in miso include *Pichia guilliermondii, Aspergillus oryzae, Zygosaccharomyces rouxii, Clavispora (Candida) lusitaniae, Candida etchellsii, Absidia corymbifera* [2, 7], most of which are not intentionally inoculated and, therefore, are likely components of the indoor microbiome. The bacteria commonly found in miso include *Bacillus amyloliquefaciens, Bacillus subtilis, Enterococcus spp., Kocuria kristinae*, *Lactobacillus plantarum, Staphylococcus gallinarum, Leuconostoc pseudomesenteroides, Staphylococcus kloosii, Tetragenococcus halophilus, Weissella cibaria, Weisella confusa, Leuconostoc citreum, Pediococcus pentosaceus and Bacillus sp.* [2, 7], most of which are not intentionally inoculated and, therefore, are also likely components of the indoor microbiome. Therefore, we suggest that future research include a more focused study relating the indoor microbiome to the succession of the miso microbiome in a single miso brewery.

## Conclusions

We have identified some of the environmental factors that contributed to variation in brewery indoor fungal and bacterial communities. These include latitude and temperature, which are not controlled directly by the brewer, and other factors over which the brewer can exert some control, including the operations that occur in the room where the miso is freely exposed to the air, and the type of interior surface on which the microbial communities develop. For example, traditional wooden surfaces, including walls, beams and miso barrels, appear to harbor different dominant fungal and bacterial taxa compared to other surfaces. The microbial communities of miso are essential to the development of the flavors, textures and nutritional properties of miso [2]. Therefore, while the Japanese Ministry of Agriculture, Forestry and Fisheries has proposed standards for miso brewing, specifying *Aspergillus oryzae* as the only fermenting microorganism and listing the major ingredients for four categories of miso [6], it may not be possible to standardize the quality of the final product without specifying operations, interior surfaces, and climate, all of which control the indoor microbiome that contributes to miso fermentation.

## Data Availability

FASTQ files are deposited in the Sequence Read Archive (SRA) at the National Center for Biotechnology Information, accession numbers PRJNA916955 and PRJNA917532.
